# Who takes care of those who care? A pilot study of psychological counseling for residents working in a paediatric hospital

**DOI:** 10.3389/fpsyg.2026.1899990

**Published:** 2026-08-28

**Authors:** Dalila Albergo, Elisa Bertolacci, Mohamad Maghnie, Cecilia Serena Pace

**Affiliations:** 1 Department of Educational Sciences (DISFOR), University of Genoa, Genoa, Italy; 2 Department of Neuroscience, Rehabilitation, Ophthalmology, Genetics, and Maternal-Child Sciences (DINOGMI), University of Genoa, Genoa, Italy; 3 Clinica Pediatrica, Endocrinologia, IRCCS Istituto Giannina Gaslini, Genoa, Italy

**Keywords:** academic engagement, counseling, paediatrics, psychological distress, residents

## Abstract

Medical residency in paediatric settings is characterized by intense emotional demands, heavy workloads, and limited opportunities for reflective processing, placing residents at increased risk for psychological distress and impaired functioning. This pilot study explored changes associated with participation in a brief psychological counseling (approximately five sessions plus follow-up, tailored to individual needs), delivered to 26 paediatric residents assessed at intake (T0), post-intervention (T1), and follow-up (T2). Participants completed self-report measures assessing psychological distress, symptoms, functioning, and academic engagement, and provided qualitative feedback through a satisfaction questionnaire. Significant improvements emerged over time in psychological well-being, symptoms, and functioning, with changes maintained at follow-up. More than half of participants reported clinically relevant distress at intake, whereas clinically significant improvement emerged only at follow-up. In contrast, academic engagement showed limited variation, with significant changes observed only in peer relationships. Qualitative feedback indicated high satisfaction with the service, perceived usefulness in managing emotional and professional challenges, and a desire for longer or ongoing support. Overall, findings suggest that brief psychological counseling may represent a feasible and potentially beneficial resource for paediatric residents and was associated with improvements in psychological well-being and reflective functioning within high-intensity clinical environments.

## 1 Introduction

### 1.1 Mental health of medical residents in paediatric context

Psychological distress among medical trainees is a major public health concern (Frishman et al., 2021). International evidence consistently shows higher rates of depressive and anxiety symptoms in medical students compared to both the general population and non-medical university peers (Stirparo et al., 2024; Rotenstein et al., 2016). This vulnerability is not limited to clinical disorders, as subclinical depressive symptoms, anxiety, emotional exhaustion, and reduced quality of life frequently emerge early during medical training (Dyrbye et al., 2006).

If undergraduate medical education is associated with increased psychological vulnerability, postgraduates training represents an additional critical period (Slavin et al., 2024). Residency involves progressive clinical responsibility, prolonged working hours, shift schedules, sleep deprivation, and sustained exposure to emotionally demanding situations (IsHak et al., 2009). These structural and relational stressors are consistently associated with reduced well-being, burnout, depressive symptoms, and anxiety disorders (Dyrbye et al., 2014). The implications extend beyond individual suffering, affecting quality of care, impaired professional functioning, and increased self-reported medical errors, underscoring the systemic relevance of residents’ mental health (West et al., 2006).

Within postgraduate medical training, paediatric-related specialties (e.g., Paediatrics, Paediatric Surgery, Child Neuropsychiatry), may entail heightened strain and fatigue. Indeed, these settings involve care of severely ill children, chronic conditions, disability, and complex family dynamics. Anxiety and depressive symptoms appear especially prevalent, likely due to emotional involvement, communication with distressed caregivers, and exposure to suffering (Cellini et al., 2017).

This combination may heighten their psychological vulnerability and reinforces the relevance of supportive interventions, such as psychological counseling, to promote well-being and professional functioning.

### 1.2 Academic engagement of medical residents in paediatric context

Although qualified physicians, medical residents remain embedded in advanced university training pathways. Their dual role as learners and practitioners makes academic engagement a central dimension of their professional and educational development. Academic engagement refers to cognitive, emotional, and behavioral investment in training, supporting motivation, persistence, and sense of belonging (Freda et al., 2023; Schaufeli et al., 2002).

In residency, particularly in paediatric contexts, academic engagement is particularly relevant due to high clinical workload, emotionally demanding encounters, and limited structured educational activities (Scaglione et al., 2025). These conditions may reduce motivation, sense of belonging, and effective integration between professional and personal life, increasing risks of disengagement and burnout.

Evidence in university populations indicates that higher academic engagement is associated with improved psychological well-being and lower distress, whereas lower academic engagement predicts dropout intentions and poorer functioning (Caldarelli et al., 2025; Greco et al., 2025b). Moreover, relational dimensions, such as meaningful connections with peers and supportive interactions with supervisors, emerge as protective factors, fostering resilience and academic persistence (Martino et al., 2026; Passeggia et al., 2026).

Although evidence on postgraduate medical trainees remains limited, preliminary findings suggest similar dynamics: more engaged residents express higher training satisfaction, better general health, and fewer episodes of excessive workload stress. In paediatric contexts, where emotional burden is heightened, academic engagement may be especially sensitive to fluctuations in psychological well-being (Cellini et al., 2017).

### 1.3 Can psychological counseling help medical residents in paediatric context?

University psychological counseling is generally conceived as brief, flexible, and free of charge intervention, with a facilitated access, aimed at supporting students experiencing mild psychological distress and academic difficulties by strengthening coping strategies and personal resources (Pace et al., 2022). Evidence suggests that these interventions improve psychological well-being and functioning, reduce anxiety and depressive symptoms, positively influence academic self-efficacy, study abilities, integration and academic engagement, and promoting students mental health (Antunes-Alves and Langmuir, 2021; Bani et al., 2022; Fortunato et al., 2026; Greco et al., 2025a,b; Pizzo et al., 2025; Scruggs et al., 2023). A recent systematic review and meta-analysis based on CORE measures (Collins et al., 2025) found consistent reductions in psychological distress following counseling interventions across higher education settings.

Even in medical students, counseling programs significantly reduce perceived stress and general psychological distress, increase mindfulness, improve dysregulation, depression and anxiety symptoms, academic motivation and psychological distress (Bani et al., 2024; Dederichs et al., 2020; Phang et al., 2016; Rajiah and Saravanan, 2014; Thompson et al., 2010).

Among medical residents, psychological interventions -e.g. short-term psychotherapy (Zisook et al., 2025)-mainly target depressive and anxiety symptoms, significantly reducing burnout levels and negative emotional states, and positively impacting their well-being and quality of life (Golob et al., 2018; Major et al., 2021; Wang et al., 2021).

More recently, interventions targeting physician well-being have expanded beyond individual counseling to include organizational strategies, peer-support initiatives, workload management programs, protected time for reflection, and institutional policies aimed at reducing burnout and promoting professional sustainability (Slavin et al., 2024; West et al., 2016). Meta-analytic evidence suggests that both individual- and organization-directed interventions are associated with improvements in physician well-being, with system-level changes often showing larger effects (Panagioti et al., 2017; West et al., 2016).

Despite these promising effects among medical residents (Bursch et al., 2017; McKinley et al., 2017; Taylor et al., 2016), specific evidence on paediatric residents remains limited, particularly regarding the integration of structured psychological counseling within residency programs in Italian academic medical settings. Most studies on this population have mainly examined burnout prevention programs, mindfulness interventions, or educational support strategies rather than structured psychological counseling services. Moreover, few studies have included follow-up assessments, limiting understanding of the durability of intervention effects over time. Our study addresses these gaps by evaluating the mid-term trajectory of the benefits of counseling interventions in terms of reducing psychological distress and improving academic engagement among residents in paediatric settings.

### 1.4 The current study

In response to growing concerns regarding students’ mental health, in Genoa at University of Genoa, the PRISMA and PRISMA 2.0 projects were developed to promote psychological well-being and prevent distress in university students through an integrated framework, combining research, psychological counseling, prevention, and training. Within PRISMA project, from December 2024 a psychological counseling service was introduced at the IRCCS Giannina Gaslini, a highly specialized and leading paediatric hospital in Italy and Europe, where clinical demands coexist with responsibilities and academic training requirements.

The present pilot study investigated whether participation in the psychological counseling program was associated with reduced self-reported psychological distress and symptoms, and with increased academic engagement, among medical residents at the IRCCS Giannina Gaslini. Given the exploratory nature of the study, it was not intended to establish causal relationships between counseling participation and changes over time, but rather to assess the trajectory of the participants’ variations. For this purpose data were collected at baseline (T0), post intervention (T1) and follow-up (T2), allowing assessment of the short-term stability of changes, which remains underexplored in this population. A secondary aim was to explore acceptability of the service through qualitative analysis of participants’ feedback.

## 2 Method

### 2.1 The PRISMA psychological counseling

The intervention consisted of brief psychological counseling, a time-limited, goal-oriented approach (Vermeulen-Oskam et al., 2024) based on an integrative psychodynamic model that supported paediatric residents in addressing professional and academic challenges by fostering self-reflection, emotional processing, reflective functioning, and adaptive coping (Wampold, 2015).

The counselor had completed a four-year postgraduate specialization in psychodynamic psychotherapy with an analytic-transactional orientation (Berne, 1961) and was undertaking advanced training in integrative psychotherapy.

Residents accessed the PRISMA psychological counseling service by completing an online request form. The intervention comprised approximately five to eight 50-min sessions, generally scheduled on a biweekly basis, with the number of sessions tailored to participants’ needs, followed by a follow-up session. Sessions were delivered either in person at the IRCCS Giannina Gaslini or online.

The intervention followed a semi-structured three-phase protocol (assessment, focused clinical work, and consolidation) based on established brief counseling models (Egan, 2013; Nelson-Jones, 2014; Stewart and Joines, 2012) and integrated within a psychodynamic, transactional analytic, and relational framework. The number of sessions varied according to participants’ clinical progression and completion of the treatment goals, with a follow-up session offered to review outcomes and maintenance strategies. Intervention consistency was ensured through the shared protocol, predefined clinical objectives aligned with the CORE-OM and SAES domains. Furthermore, methodological coherence was ensured through monthly supervision with a senior psychodynamic supervisor, bimonthly PRISMA team intervision meetings and hoc consultation with the PRISMA director for more complex cases.

The counseling was offered free of charge and addressed to postgraduate residents in Paediatrics, Child Neuropsychiatry, and Paediatric Surgery, as well as students enrolled in degree programs in Paediatric Nursing, Neuro and Psychomotor Therapy of Childhood, and doctoral programs in Paediatric Sciences. For the purposes of this report only the data from postgraduate residents were included.

### 2.2 Participants and procedures

Since its establishment, 48 individuals have been referred to the psychological counseling service. Among them, 36 postgraduate medical residents accessed the counseling intervention between November 2024 and April 2026 (22% males; mean age ± SD = 28.56 ± 3.35 years), enrolled in residency programs in Paediatrics (*n* = 24), Child Neuropsychiatry (*n* = 10), and Paediatric Surgery (*n* = 2). At the time of data analysis, 26 residents had completed all assessment time points, including baseline (T0), post-intervention (T1), and follow-up (T2), and were therefore included in the analyses. For the remaining ten residents -except one who dropped-out- follow-up data were not yet available.

The study was approved by the Ethics Committee of the University of Genoa (protocol n. 2023/85) and written informed consent was obtained from all participants prior to data collection.

### 2.3 Measures and variables

Participants completed a brief socio-demographic questionnaire including age, gender and course of study in which they were enrolled. In line with the monitoring activities of the national network of Academic Psychological Counseling Services (SCPA), the following self-report measures were administered to participants:

The Italian validated version of the *Clinical Outcomes in Routine Evaluation–Outcome Measure* (CORE-OM; Evans et al., 2000; Palmieri et al., 2009) was used to assess psychological distress and well-being. The CORE-OM is a 34-item self-report questionnaire comprising four subscales: Well-being (4 items), Symptoms (12 items), Functioning (12 items), and Risk to self and others (6 items). Items refer to the previous week and are rated on a 5-point Likert scale ranging from 0 (not at all) to 4 (most or all the time). Scores for the subscales and the total score were calculated as mean values, with higher scores indicating greater psychological distress. In the present sample, internal consistency was satisfactory for all domains (Cronbach’s *α* > 0.70) and excellent for the total score (Cronbach’s α > 0.90).

The *SInAPSi Academic Engagement Scale* (SAES; Freda et al., 2023) is a 29-item self-report measure developed and validated in Italy to assess academic engagement and psychological resources related to academic demands. The questionnaire comprises six dimensions: university value and sense of belonging (6 items), persistence in the university choice (4 items), course value (7 items), relationships with professors (4 items), relationships with peers (5 items), and integration between the university and relational network (3 items). Items are rated on a 5-point Likert scale ranging from 1 (not at all) to 5 (completely). Subscale and total scores were calculated as mean values, with higher scores indicating greater academic engagement. In the present sample, all dimensions showed satisfactory internal consistency (Cronbach’s α > 0.75).

Participants completed a *Satisfaction Questionnaire* (see Supplementary material) developed by the University Center for Psychological Clinical Services (SCUP) of the University of Padua to assess satisfaction with the psychological counseling service. The questionnaire evaluates several aspects of the intervention on a 5-point Likert scale (1 = not at all, 5 = very much), including service accessibility, therapist availability, quality of listening and communication, perceived effectiveness of the strategies used, facility comfort, privacy, quality of care, and overall satisfaction. An open-ended item was also included to collect participants’ suggestions and feedback.

### 2.4 Data analysis

Descriptive statistics were computed for all study variables. Data analyses were conducted using JASP (JASP Team, 2025). Given the small sample size and the non-normal distribution of some variables, changes in CORE-OM and SAES scores across T0, T1, and T2 were evaluated using non-parametric Friedman tests. Significant effects were further explored through Conover *post hoc* pairwise comparisons with Bonferroni correction.

To complement the analysis of mean score changes, individual-level reliable change (RC) and clinically significant change (CSC) were examined according to the established CORE-OM criteria for the Italian population (Palmieri et al., 2009).

## 3 Results

### 3.1 Descriptive results

Participants (*N* = 26, 23% males, mean age ± SD = 28.81 ± 3.61 years) were enrolled in residency programs in Paediatrics (*n* = 17), Child Neuropsychiatry (*n* = 8), and Paediatric Surgery (*n* = 1).

Most participants accessed the counseling intervention primarily for personal reasons (84.6%) rather than academic concerns (15.4%). Participants completed three–eight sessions (M = 6.15). The counseling program lasted an average of 3.96 months, and follow-up assessments were conducted a mean of 6.95 months after post-intervention assessment. The intervention was delivered almost entirely in person, with only occasional online sessions (6.88% of total number).

### 3.2 Changes in CORE-OM and SAES scores across assessment points

Descriptive statistics of both CORE-OM and SAES scores are shown in Table 1.

**Table 1:** Means and standard deviations for all study variables across the assessment points.

Measure	T0 Mean (SD)	T1 Mean (SD)	T2 Mean (SD)
CORE-OM			
Well-being	2.01 (0.86)	1.64 (0.79)	1.31 (0.83)
Symptoms	1.61 (0.92)	1.24 (0.84)	0.96 (0.62)
Functioning	1.38 (0.62)	1.14 (0.47)	1.04 (0.50)
Risk	0.06 (0.20)	0.03 (0.06)	0.01 (0.05)
Total	1.30 (0.62)	1.04 (0.52)	0.86 (0.45)
SAES			
Belonging	4.24 (0.85)	4.37 (0.70)	4.45 (0.61)
Persistence	4.12 (0.96)	4.19 (0.72)	4.19 (0.72)
Course value	4.19 (0.83)	4.10 (0.82)	4.23 (0.67)
Professor relationships	2.83 (0.80)	2.77 (0.85)	2.78 (0.76)
Peers relationships	3.85 (0.79)	3.79 (0.80)	4.09 (0.94)
Integration	3.86 (1.10)	3.96 (0.99)	4.00 (1.02)

T0 = intake, T1 = intervention conclusion, T2 = follow-up.

**Figure 1:**
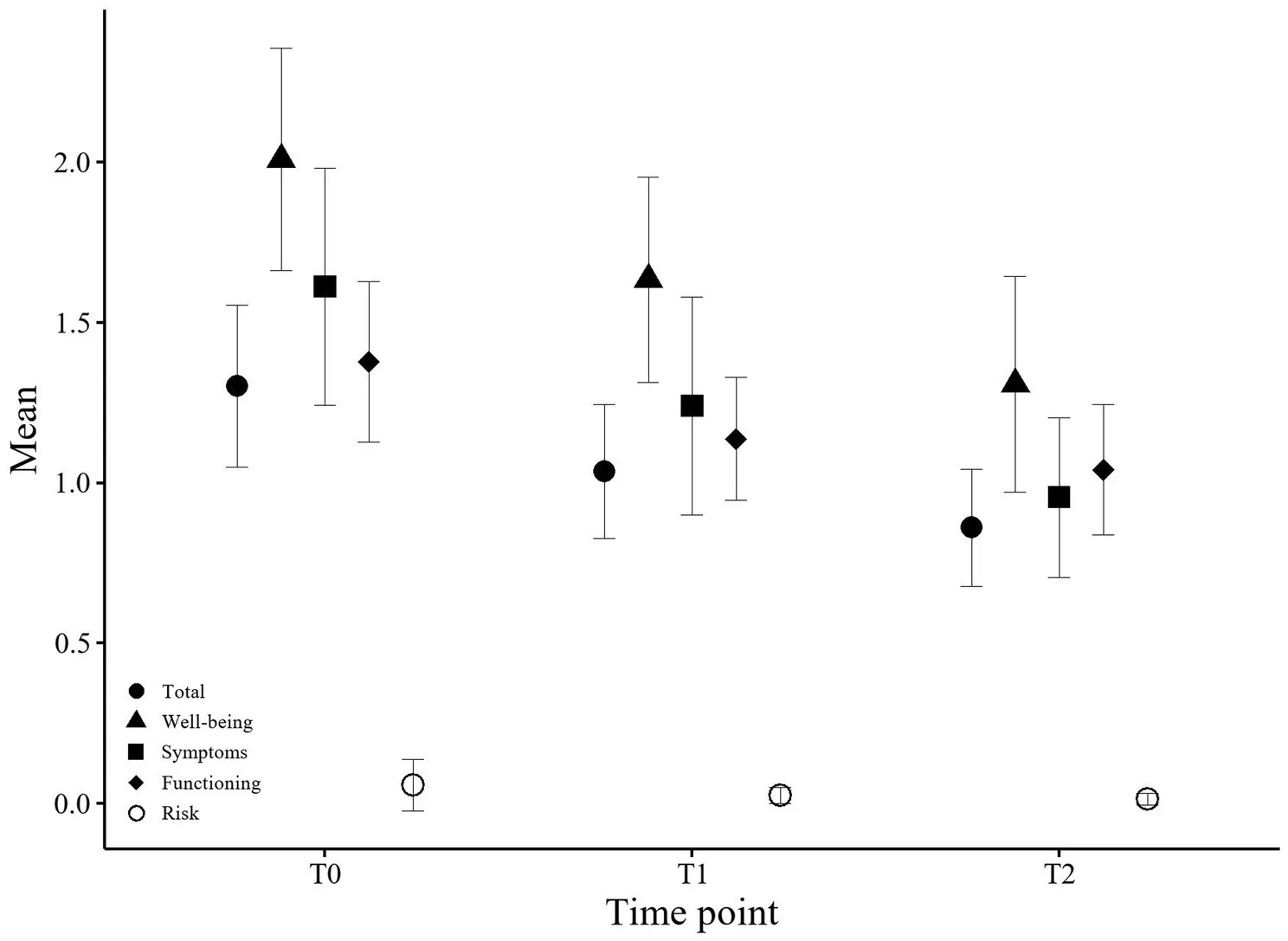
CORE-OM mean scores across the assessment points: T0 = intake, T1 = intervention conclusion, T2 = follow-up.

The Friedman test showed significant effects of time for all CORE-OM subscales except for risk (χ^2^_(2, 50)_ = 2.00, *p* = 0.37, W = 0.04). Specifically, well-being improved over time, χ^2^_(2, 50)_ = 8.63, *p* = 0.013, W = 0.17, with post-hoc tests indicating a significant score decrease from T0 to T2 (*p* = 0.009). Symptoms also decreased significantly, χ^2^_(2, 50)_ = 18.59, *p* < 0.001, W = 0.36, with significant T0–T1 (*p* = 0.002) and T0–T2 contrasts (*p* < 0.001). Functioning showed a similar pattern, χ^2^_(2, 50)_ = 6.88, *p* = 0.032, W = 0.13, with significant improvement from T0 to T2 (*p* = 0.032). The total score decreased significantly over time, χ^2^_(2, 50)_ = 12.87, *p* = 0.002, W = 0.25, with post-hoc tests again showing significant reductions from T0 to T1 (*p* = 0.021) and T0 to T2 (*p* < 0.001; see Figure 1).

At baseline (T0), 54% of participants reported CORE-OM scores above the clinical threshold. Reliable changes (RC) and clinically significant changes (CSC) in CORE-OM total scores are shown in Table 2.

**Table 2:** Reliable change (RC) and clinically significant change (CSC) in CORE-OM total scores across the assessment points.

Change index	T1 vs. T0 *n* (%)	T2 vs. T0 *n* (%)
Positive RC	11 (42%)	8 (31%)
Negative RC	2 (8%)	1 (4%)
CSC	0 (0%)	3 (12%)

T0 = intake, T1 = intervention conclusion, T2 = follow-up.

**Figure 2:**
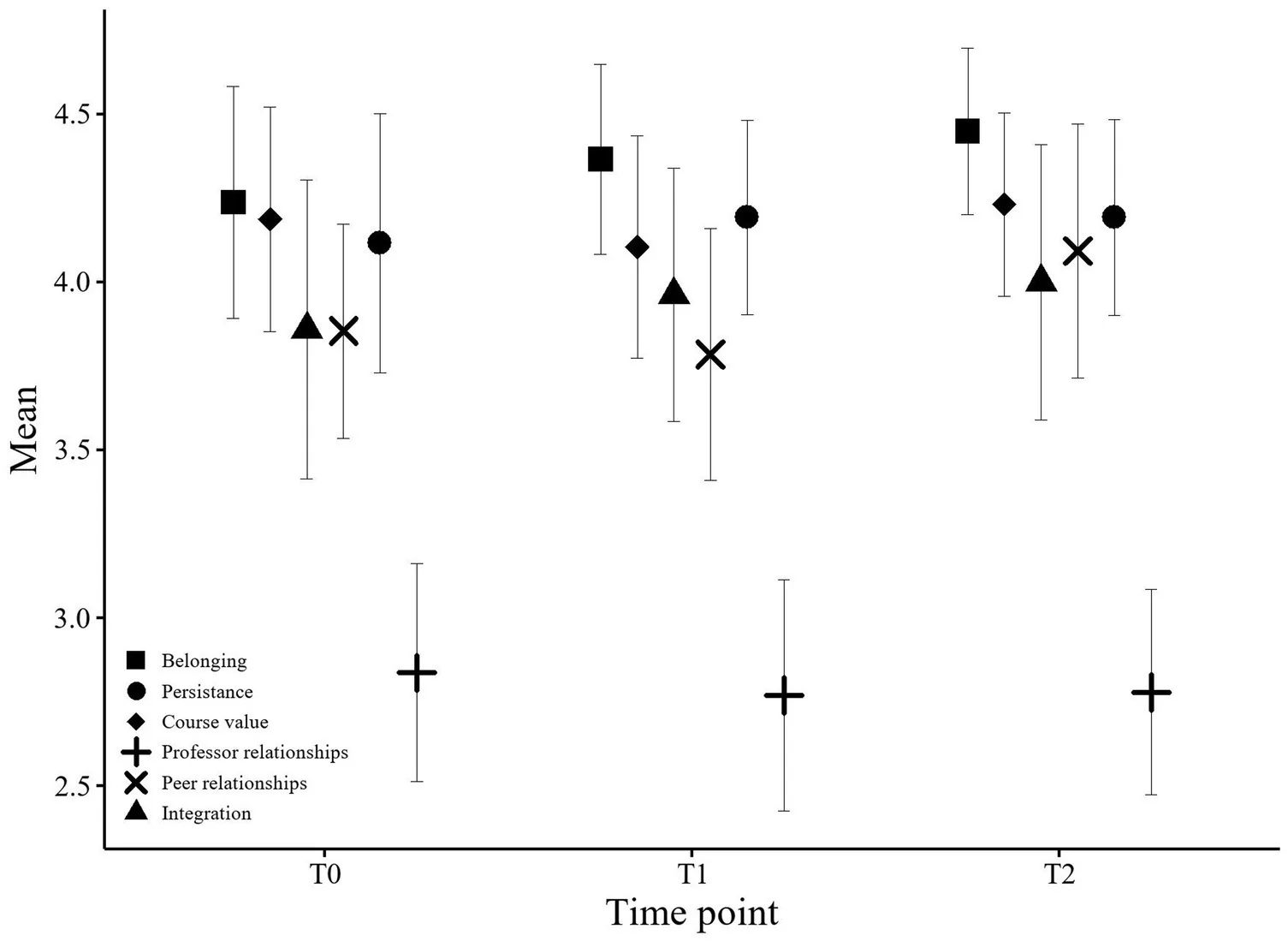
SAES mean scores across the assessment points: T0 = intake, T1 = intervention 194 conclusion, T2 = follow-up.

SAES subscales (Belonging, Persistence, Course value, Professor relationships, Integration) showed slightly increasing mean scores across time. However, only Peers relationships showed significant differences, χ^2^_(2, 50)_ = 6.26, *p* = 0.044, W = 0.12, with a significant score increase from T1 to T2 (*p* = 0.044; Figure 2).

### 3.3 Satisfaction questionnaire

The analysis of the satisfaction questionnaire completed by the users provides an overall highly positive evaluation of the counseling service.

Most participants reported feeling welcomed (M = 4.85 ± 0.46 out of 5), described the experience as very useful (M = 4.46 ± 0.99 out of 5), reported that counselor was able to understand their problem (M = 4.69 ± 0.62 out of 5), and overall satisfaction with the psychological counseling was very high (M = 4.62 ± 0.75 out of 5) with 73.1% of residents scoring 5 and 19.2% scoring 4. Otherwise, regarding the perception of having addressed the initial concern that led them to the service, the majority reported a satisfactory or partially satisfactory outcome (M = 3.50 ± 1.07 out of 5), a result consistent with the structural characteristics of brief psychological counseling, which does not allow for the in-depth exploration of more complex issues. Indeed, in some cases, counseling primarily serves to fostering participants’ awareness and facilitating access to more structured interventions, such as psychotherapy.

In the suggestions collected, many users emphasized the usefulness of the service, described as “*precious*”: “*It would be very nice if the service could continue in the long term because it helped me a lot to live my specialty in a better way*.” Moreover, some participants expressed the wish for an increased number of sessions or a longer overall duration, as well as the desire for the counseling service to become a stable and continuous resource. A focus was given to the specific context the intervention was given: *“It should be a service guaranteed on a continuous basis especially for healthcare specialists within the IRCCS since are exposed to very disturbing contents and experiences […],”* thus highlighting the peculiar needs that the population analyzed in this study have.

## 4 Discussion

Consistent with our expectations, participants showed significant improvements across most CORE-OM domains from baseline to post-intervention and follow-up, indicating reduced psychological distress, symptoms, and functional difficulties. Effect size estimates suggested small to medium effects, with the largest effect observed for the symptoms’ reduction, supporting the clinical relevance of the observed changes despite the limited sample size.

These findings are consistent with previous research demonstrating the effectiveness of psychological counseling and supportive interventions in reducing emotional distress, anxiety, depressive symptoms, and burnout among university students and medical trainees (Cerolini et al., 2023; Golob et al., 2018; Major et al., 2021; Wang et al., 2021). The maintenance of these improvements at follow-up may suggest benefits extending beyond the intervention period; however, given the absence of a control group, alternative explanations such as spontaneous adaptation, regression to the mean, and contextual factors cannot be excluded. Extending previous evidence to paediatric residents, our findings suggest that brief counseling may represent a useful opportunity for emotional processing and coping with the specific demands of paediatric clinical training. No significant changes emerged in the Risk domain, likely because baseline scores were already low, suggesting limited levels of self-harm and risk-related concerns among participants at intake.

Individual-level analyses indicated that more than half of participants exceeded the clinical threshold at baseline, suggesting clinically relevant distress among residents accessing counseling. Reliable improvements emerged in a proportion of participants, whereas clinically significant change was observed only at follow-up. Given the brief nature of the intervention, this finding may indicate that meaningful changes require time to consolidate, particularly in domains such as emotional regulation and coping.

Importantly, descriptive information regarding residents’ presenting concerns helps contextualize these quantitative improvements. Participants frequently sought support for difficulties in balancing professional and personal life, managing performance anxiety, coping with criticism and expectations from senior physicians, and processing emotionally demanding clinical experiences. Counseling sessions frequently addressed themes related to emotional regulation, assertive communication, and reflective capacities in response to stressful interpersonal dynamics within clinical teams.

In contrast, the counseling intervention was not associated with significant improvements in most dimensions of academic engagement. Although SAES scores showed slight positive trends, a significant increase emerged only for peer relationships. This finding differs from studies conducted with university students, in which counseling has been associated with improvements in academic functioning and engagement (Antunes-Alves and Langmuir, 2021; Bani et al., 2024). However, SAES scores were already high at baseline, particularly for belonging, persistence, and course value, suggesting a possible ceiling effect. Moreover, the small effect size observed for peer relationships indicates that, although statistically significant, the magnitude of change was limited and may reflect a gradual improvement in interpersonal connectedness rather than a broader modification of academic engagement.

Furthermore, the demanding nature of paediatric residency may limit the extent to which a brief intervention can influence academic engagement within a relatively short period. While reductions in psychological distress may facilitate motivation and participation over time (Slavin et al., 2024), residents’ daily experiences are primarily shaped by clinical responsibilities and professional identity development rather than academic activities. This may also explain why peer relationships represented the only domain showing significant improvement at follow-up. The delayed nature of this effect suggests that changes in interpersonal functioning may require more time to consolidate than symptom reduction. The small effect size further supports the interpretation that interpersonal changes may represent an emerging process rather than an immediate outcome of the intervention. Given the central role of peer support in coping with clinical and emotional demands, it is possible that counseling contributed to strengthen residents’ capacity to share difficulties, communicate more openly, and experience greater connectedness within the clinical team. This interpretation is consistent with the clinical themes frequently addressed during the intervention, including interpersonal communication, emotional regulation, and the management of feedback and criticism within hierarchical medical environments. Finally, the SAES may be less sensitive to change in residency populations, whose engagement is expressed primarily through clinical practice and team membership rather than through traditional student-centered academic activities. Furthermore, some dimensions of academic engagement, such as sense of belonging, persistence, and perceived course value, may represent relatively stable aspects of residents’ educational experience and therefore require longer observation periods or more intensive interventions to show measurable changes. In postgraduate medical training, engagement is also embedded within organizational and clinical contexts that may evolve more slowly than individual psychological distress.

### 4.1 Strengths and limitations

This study has several strengths. First, it focuses on a highly specific and under-researched population: paediatric residents working in a high-intensity hospital setting. Second, the inclusion of a follow-up assessment allowed us to examine the maintenance of changes over time. Third, validated instruments with good psychometric properties were used to assess both psychological distress and academic engagement.

Several limitations should also be acknowledged. The small sample size and the recruitment of participants from a single institution (IRCCS Istituto Giannina Gaslini) limit the generalizability of the findings and reduce statistical power. Nevertheless, the estimation of effect sizes provides additional information beyond statistical significance and suggests that the observed changes, particularly in symptom reduction, reached a meaningful magnitude despite the limited statistical power. Furthermore, the absence of a control group precludes causal inferences regarding intervention effectiveness: because of the exploratory pre-post design and the lack of a comparison group, the findings should be interpreted as temporal associations rather than evidence of intervention effectiveness. Moreover, the exclusive use of self-report measures may have introduced response biases. Outcomes may also have been influenced by unmeasured factors, such as personality characteristics, coping styles, and contextual stressors. Finally, self-selection bias and participant attrition may have affected the results, as residents voluntarily accessed the counseling service and not all participants completed the follow-up assessments.

### 4.2 Conclusion and future directions

Overall, this pilot study provides preliminary evidence that brief psychological counseling may represent a feasible and beneficial resource for paediatric residents working in emotionally demanding clinical environments. Participation in the counseling service was associated with sustained reductions in psychological distress, symptoms, and functional difficulties, with small-to-medium effect sizes indicating preliminary evidence of clinically meaningful change, whereas no significant changes emerged in risk-related experiences and in most dimensions of academic engagement, possibly reflecting both the specific characteristics of residency training and the limited sensitivity of student-oriented measures in this population. High levels of participant satisfaction, together with requests for longer or ongoing support, further suggest that residents perceived the intervention as meaningful and relevant to their professional and personal needs.

Future research should replicate these findings using larger samples and controlled designs, while exploring the mechanisms through which counseling exerts its effects and its potential integration with structured reflective practice. Longitudinal studies are also needed to determine whether improvements in psychological well-being translate into benefits for professional development, clinical functioning, and retention in paediatric specialties. Notably, residents frequently had to reschedule or cancel appointments because of urgent clinical responsibilities, highlighting workload-related barriers to accessing psychological support. Future programs may therefore benefit from greater scheduling flexibility and stronger institutional recognition of psychological well-being as an integral component of residency training.

## Data Availability

The raw data supporting the conclusions of this article will be made available by the authors, without undue reservation.
