# Supplementary material for: Who takes care of those who care? A pilot study of psychological counseling for residents working in a paediatric hospital
Source: Front Psychol. 2026 Aug 28;17:1899990. doi: 10.3389/fpsyg.2026.1899990 (PMC13561836; doi:10.3389/fpsyg.2026.1899990)
Supplement: Supplementary file 1 [file Supplementary_file_1.DOCX]

Supplementary Material

**Satisfaction Questionnaire**

Instructions: we now ask you to think to your experience at counseling and answer honestly the following questions considering the scale below: from 1 = NOT AT ALL to 5 = VERY MUCH.

1. The room seemed cozy, comfortable and clean
2. Environments have ensured confidentiality
3. Health safety aspects have been taken care of
4. I felt treated with care and politeness
5. Reception was adequate
6. The information received was clear and understandable
7. The information received was complete
8. The Psychologist has met the appointment schedule
9. The Psychologist has understood the problem
10. The experience has been useful
11. Do you think you have solved the problem you have addressed to the service?
12. Agreed objectives have been achieved

Please rate your overall satisfaction with the service from 1 = VERY BAD to 5 = VERY GOOD
